# Antimicrobial resistance rates in gram-positive bacteria do not drive glycopeptides use

**DOI:** 10.1371/journal.pone.0181358

**Published:** 2017-07-20

**Authors:** Beryl Primrose Gladstone, Andrea Cona, Parichehr Shamsrizi, Tuba Vilken, Winfred V. Kern, Nisar Malek, Evelina Tacconelli

**Affiliations:** 1 Infectious Diseases, Department of Internal Medicine I, DZIF Partner, Tübingen University Hospital, Tübingen, Germany; 2 Infectious Diseases and Tropical Medicine, San Paolo Hospital, University of Milan, Milan, Italy; 3 Infectious Diseases, Department of Medicine, University Hospital and Medical Center and Albert-Ludwigs-University Faculty of Medicine, Freiburg, Germany; University Medical Center Groningen, NETHERLANDS

## Abstract

Surveillance data are considered essential to appropriate empiric antibiotic therapy and stewardship. The objective of this study was to determine if a change in the rates of antibiotic resistance impacts antibiotic use in European hospitals. Glycopeptides use was selected to study the correlation between resistance rates and antibiotic use because of the restricted spectrum against resistant gram positive bacteria. PubMed, ECDC databases and national/regional surveillance systems were searched to identify glycopeptides´ consumption in defined daily dose per 1000 inhabitant-days (DID) and rate of methicillin-resistant *Staphylococcus aureus* (MRSA), methicillin-resistant coagulase negative staphylococci (MRCoNS), and vancomycin-resistant enterococci (VRE) in bloodstream infections (BSIs) in European countries between 1997 and 2015. Time trends were studied and associations between DID and BSI resistance rates were tested using multi-level mixed effect models. To account for the gap in the publication and dissemination of the yearly resistance data, a 2-year lag in the resistance rates was applied. Data on glycopeptides´ DID and resistance rates of target microorganisms in blood cultures were identified among 31 countries over a 19-year period. Glycopeptides use significantly increased (p<0·0001) while rates of MRSA BSIs decreased in majority of the countries (p<0·0001) and MRCoNS and VRE BSIs remained stable. Variation in glycopeptides’ DID was not associated with variation in BSIs due to MRSA (p = 0·136) and VRE (p = 0·613). After adjusting for MRCoNS and VRE resistance rates, among 21 countries, 11 (52%) had a concordant and 10 (48%) a discordant trend in yearly glycopeptides´ DID and MRSA BSI rates. No correlation was found between resistance rates and DID data even among 8 countries with more than 5% decrease in MRSA rates over time. (RC -0·009, p = 0·059). Resistance rate of MRSA, MRCoNS, and VRE BSIs does not impact DID of glycopeptides in European hospitals. This finding is key to redefining the role and structure of antimicrobial surveillance and stewardship programmes.

## Introduction

Rising global resistance rates and emergence of new resistance mechanisms, along with an almost empty antibiotic drug discovery pipeline, are creating a dramatic scenario in hospitals worldwide.[1, 2] In Europe, bloodstream infections (BSIs) due to antibiotic-resistant organisms have been estimated to kill about 25,000 people and to add about 1·5 billion euros of additional healthcare costs and productivity losses every year.[3] To address this problem, multifaceted approaches, such as antimicrobial stewardship programme and antimicrobial resistance (AMR) surveillance, have been implemented in many hospitals with variable results for efficacy and costs.[2, 4–6] Essential components of these programmes are active monitoring of resistance and fostering of appropriate antimicrobial use that optimises patients´ clinical outcomes while minimising unintended consequences of antimicrobial use, including toxicity and the emergence of resistance.[7, 8]

Updated information from AMR surveillance systems plays a pivotal role in this approach. Knowledge of surveillance data should improve public health at different levels, from the local (patients’ clinical outcomes) to the more global (hospital and community AMR rates) perspective. Indeed, data from global surveillance systems should provide information on new worrisome AMR trends and allow policymakers at national and international level to design new strategies to face the threat.[9] Yearly European surveillance data are publicly available from the European Centre for Disease Prevention and Control (ECDC)[10] as well as from many national cohorts.[11–13] However, data linking the antibiotic use and availability of surveillance data of target microorganisms are not available.

We did an observational ecological study to ascertain the association between trends in antibiotic use and AMR surveillance data in hospitalised patients in European countries.

## Materials and methods

### Outcomes

Glycopeptides use was selected to study the correlation between resistance rates and antibiotic use because the restricted spectrum of these intravenous drugs covers mainly severe infections due to methicillin-resistant *Staphylococcus aureus* (MRSA), methicillin-resistant coagulase-negative staphylococci (MRCoNS) and vancomycin-susceptible enterococci (VSE). Selection of other antimicrobial classes would have involved a wider bacterial spectrum and introduced confounding in the correlation analysis.

The primary outcomes of this study were the yearly resistance rates of MRSA, MRCoNS, and VRE in BSI expressed as percentages and glycopeptides use expressed as defined daily dose (DDD) per 1000 inhabitant–days (DID). Rate of MRSA is defined as the proportion of methicillin resistance among *S*. *aureus* isolates while rate of VRE as the proportion of vancomycin resistance among enterococci isolates. When available, antibiotic use data were also collected as other measures such as recommended daily dose per 100 patient-days or DDD per 100 occupied bed-days. Measures other than DID were used only for within-country comparisons over time and not for inter-country analyses. When glycopeptides use data were not available, vancomycin data were used instead. VRE rates were used to represent VSE for consistency within the paper with MRSA and MRCoNS data.

### Study design

We performed an ecological observational study and searched yearly national surveillance data for glycopeptides´ DID and rates of BSIs due to MRSA, MRCoNS and VRE. The target population was hospitalised patients in 31 European countries over a 19 year-period (1997–2015). Data were first extracted from the European Antimicrobial Resistance Surveillance Network (EARS-Net)[14] database and the European Surveillance of Antimicrobial Consumption (ESAC). [15] To reduce potential errors in measurement we contacted experts from the European Society of Infectious Disease and Clinical Microbiology (ESCMID); we developed a standardized protocol to search online information on surveillance systems and to address experts; and interviewers (BPG and TV) were trained to ask relevant questions. For the European countries for which EARS-Net or ESAC data were not available or incomplete for the study period, a literature search using Pubmed was conducted to identify national / regional surveillance systems or cohort studies focused on BSIs surveillance and/or antibiotics use in hospitalised patients. Reports of ECDC point-prevalence surveys [10] were also searched.

### Statistical analysis

We included only countries with more than four data points of yearly rates of target microorganisms in blood cultures and glycopeptides´ DID during the study period in the analysis. The longitudinal data on resistance rates and glycopeptides usage expressed as log-transformed DID consumption were tested for time trends using linear trend analysis and we report the yearly change (YC) of resistance rates as well as the rate of yearly change (RYC) of glycopeptides´ DID assuming linearity. The overall time trend as well as inter-correlation of resistance rates and glycopeptides usage were studied using multilevel mixed-effect linear models allowing for a random variance at the yearwise and countrywise estimates to account for interdependence of data. To account for the gap in the publication and dissemination of the yearly resistance data to the medical community, a 2-year lag in the rates was applied. The regression coefficients (RC) refer to the change in log transformed glycopeptide consumption with a unit change in the rate of a specific resistant strain. Thus confounding effects of rates of other resistant strains and delay in publication were adjusted for in the analyses. Final analysis was restricted to the period from 2005 until 2015 since the downward trend of the MRSA prevalence per 1000 patient days in Europe was reported for the first time in 2005[16].

Additionally, correlation between glycopeptides`DID and resistance rates of BSIs due to MRSA, MRCoNS, and VRE were classified as discordant or concordant trends on the basis of the direction of the relative change in glycopeptides`DID and in the BSI resistance rates for each European country. Pairwise deletion of missing data was performed. The statistical analyses were conducted using STATA version 14.0.[17]

## Results and discussion

Overall, we mapped 13 surveillance systems addressing resistance among targeted bacteria and antibiotic consumption (S1 Table). Relevant data (S1 File) were extracted for 31 countries from 6 surveillance systems. Incomplete or partial data from the EARS and ESAC databases were integrated with 4 national or regional surveillance systems. Table 1 illustrates the main characteristics of the surveillance systems.

**Table 1:** National surveillance systems and European research projects reporting surveillance data on bloodstream infections (BSIs) due to methicillin-resistant Staphylococcus aureus (MRSA), methicillin-resistant coagulase negative staphylococci (MRCoNS), and vancomycin-resistant enterococci (VRE) included in the study.

Surveillance system	Acronym	Country	Coverage
European Antimicrobial Resistance Surveillance Network**[14]**	EARS-Net	Europe	30 European countries
European Surveillance of Antimicrobial Consumption**[15]**	ESAC	Europe	30 European countries
English surveillance programme for antimicrobial utilisation and resistance**[12]**	ESPAUR	England	160 national acute-care hospitals
Observatoire National de l’Épidémiologie de la Résistance Bactérienne aux Antibiotiques**[13]**	ONERBA	France	15 microbiological networks
Surveillance of Health Care Associated Infections in Catalonia**[18]**	VINCat	Catalona (Spain)	54 hospitals
Surveillance Project for Antibiotic Use in German Acute Care Hospitals (Bunderverhand Deutscher Krankenhaus Apotheker e.V) **[19]**	RKI-if-ADKA	Germany	109 acute-care hospital centres

### MRSA-BSI

Data on MRSA rate could be retrieved from the EARS-Net for 28 countries while incomplete data were retrieved for Croatia (data available only from 2010 onwards) and Slovakia (missing data from 2006 to 2010). Data were not available for one country (Liechtenstein).

Over the study period, MRSA rate significantly decreased (YC: -0·510; p<0·0001) ranging from 0% to 65% of *S*. *aureus* BSI. Iceland, Norway, Sweden, Netherlands, Denmark, and Finland had resistance rate of <2% while Slovakia, Cyprus, Italy, Greece, Portugal, Malta, and Romania had resistance rates of >25%. Correlation coefficients showed a significant decreasing trend in MRSA rates in Austria (p = 0.045), Belgium (p<0·0001), France (p<0·0001), Germany (p = 0·0006), Greece (p = 0.029), Ireland (p<0·0001), Latvia (p = 0·0047), Malta (p = 0·0092), Spain (p = 0·024), and the UK (p<0·0001). Except for increasing trend in Slovakia and a subtle increase in Norway, MRSA rates either decreased (although not significantly) or remained stable in the other countries. Table 2 displays trends per analysed country.

**Table 2:** Time trends of rates (%) of methicillin resistance among Staphylococcus aureus and vancomycin resistance among enterococci isolates from bloodstream infection (BSI) and glycopeptides use expressed as Defined Daily Dose per 1000 inhabitant–days (DID) for European countries during 11-year period (2005 to 2015).

	MRSA		VRE			Glycopeptide consumption (GC) (expressed in DID)*	
	rates		rates				
	(%)		(%)				
Country	Yearly change	P	Yearly change	P value	Annual change in log GC	Rate of yearlychange (RYC)	P
		Value					Value
	
Austria	-0.377	0.0445	-0.374	0.0108			
Belgium	-1.455	0.0001	0.646	0.0757	-0.025	0.975	0.0012
Bulgaria	-0.885	0.0886	-1.147	0.0155	0.127	1.135	<0.0001
Croatia	-0.700	0.2998	-3.163	0.1593	0.068	1.070	0.2074
Cyprus	-1.015	0.1826	-0.077	0.9562			
Czech Republic	0.047	0.5305	0.031	0.9221			
Denmark	0.038	0.426	-0.387	0.0011	0.064	1.066	0.0009
Estonia	-0.025	0.9216	0.086	0.2854	0.097	1.102	0.0001
Finland	-0.067	0.2461	0.027	0.0629	0.008	1.008	0.3851
France	-1.219	<0.0001	0.200	0.0178	-0.003	0.997	0.3312
Germany	-0.913	0.0006	-0.276	0.4436	0.067	1.069	0.0074
Greece	-0.555	0.0288	2.008	0.0016	-0.010	0.990	0.0632
Hungary	0.178	0.5467	-1.218	0.003	0.052	1.054	0.0077
Iceland	-0.059	0.8027	-0.041	0.8946			
Ireland	-2.628	<0.0001	-1.413	0.0001	0.119	1.127	0.0017
Italy	-0.209	0.2763	0.891	0.0912	0.140	1.150	0.0629
Latvia	-1.075	0.0047	-1.385	0.0336	0.032	1.032	0.0424
Lithuania	-0.429	0.1505	-0.995	0.1092	0.095	1.100	0.3302
Luxembourg	-0.600	0.161	0.175	0.897	-0.013	0.987	0.2036
Malta	-1.472	0.0092	0.027	0.1215	0.070	1.072	0.0017
Netherlands	0.015	0.6832	-0.067	0.0197	0.033	1.034	0.0001
Norway	0.098	0.0046	-0.079	0.2618	0.081	1.085	<0.0001
Poland	-0.038	0.9367	-1.878	0.0007	-0.123		.
Portugal	0.006	0.9845	1.074	0.0013	0.010	1.010	0.2997
Romania	1.496	0.2319	-2.336	0.004			
Slovakia	1.194	0.0099	-1.246	0.0187	0.146	1.157	<0.0001
Slovenia	0.221	0.2609	0.329	0.383	0.024	1.024	0.0014
Spain	-0.341	0.0242	0.104	0.2855	0.063	1.065	0.1171
Sweden	0.008	0.6915	0.054	0.2762	0.039	1.040	0.0001
United Kingdom	-3.634	<0.0001	0.862	0.2462	0.090	1.095	0.0011

* For Spain and Germany, glycopeptides consumption data presented as DDD per 100 occupied bed days and recommended daily dosage per 100 patient-days respectively

### MRCoNS BSI

The EARS-Net database does not report data on the rate of BSI due to CoNS. Literature search identified one national surveillance system from France (ONERBA; Observatoire National de l’Épidémiologie de la Résistance Bactérienne aux Antibiotiques) reporting yearly prevalence data from 1996 to 2011. Analysis of these data showed a stable trend of BSI due to MR-CoNS.

In order to increase representativeness of data, we searched the ECDC point-prevalence surveys that provided data on the resistance rate of CoNS-BSI in intensive care unit (ICU) for 15 countries at three time points: 2004–2005, 2007, and 2012. At least two time points were available for ten countries (Austria, Belgium, France, Germany, Italy, Lithuania, Luxembourg, Portugal, Slovakia, and Spain). Since ICU BSIs are a primary contributor to CoNS infections and a high proportion of CoNS BSI (> 80%) are resistant to methicillin, these data were used as surrogate data to represent the rate of MRCoNS BSIs in hospitalized patients.

The overall rate of CoNS among ICU BSIs in Europe remained stable being 29·4% in 2004–2005, 28·5% in 2007, and 22·3% in 2012. Except for Lithuania and Austria, which had a non-significant increase in this period, the data showed a non-significant decreasing trend from 2004 to 2012 in the other countries.

### VRE BSI

VRE rate data could be retrieved from the EARS-Net for 28 countries while incomplete data were retrieved for Croatia (data available only from 2010 onwards) and Slovakia (missing data from 2006 to 2010). Data were not available for one country (Liechtenstein).

During the study period, the overall rate of VRE slightly increased (YC 0.2055, p = 0.046) ranging from 0·5% to 43%. Ireland, the UK, Cyprus, Greece, and Portugal reported a resistance rate of >15% and 15 countries had a resistance rate of < 5%. While France (p = 0·018), Greece (p = 0.002), and Portugal (p = 0·001) showed a significant decreasing linear trend, Austria (p = 0·012), Bulgaria (p = 0·016), Denmark (p = 0·001), Hungary (p = 0·003), Ireland (p = 0·0001), Latvia (p = 0·034), Netherlands (p = 0·020), Poland (p = 0·0007), Romania (p = 0·004), and Slovakia (p = 0·019) displayed a significant increasing trend between 2005 and 2015. Croatia and Lithuania showed an increasing though not significant trend (p = 0·159 and p = 0·109 respectively).

### Glycopeptides´ DID

Glycopeptides use data expressed as DID could be retrieved from the ESAC database for 23 countries. Due to the lack of national available data, the following regional data were used: the English Surveillance Programme for Antimicrobial Utilisation and Resistance (ESPAUR) 2014 report for UK[12]; the Catalonia surveillance data for Spain [20] and the RKI-if-ADKA GERMAP (Antibiotic use and the spread of antibiotic resistance in human and veterinary medicine in Germany) for Germany.[19] Data were not available for five countries (Austria, Cyprus, Czech Republic, Iceland, and Liechtenstein). Overall, at least four time points were available for 24 countries.

During the study period, glycopeptides´ DID significantly increased at a rate of 1.058 (p<0·0001) ranging from 0.975 in Belgium to 1.150 in Italy per year. Ireland, Greece, and Italy had the highest use (> 0.05 DID) while Bulgaria, Estonia, and Norway had the lowest use (< 0.015 DID). Table 2 displays trends per analysed country.

In Spain and Germany, glycopeptides use data were presented as DDD per 100 occupied bed days and recommended daily dosage (RDD) per 100 patient days respectively. Measures other than DID were used only for within-country comparisons over time and showed an increasing trend for both countries.

### Glycopeptides´ DID and BSI resistance rates

**Fig 1:**
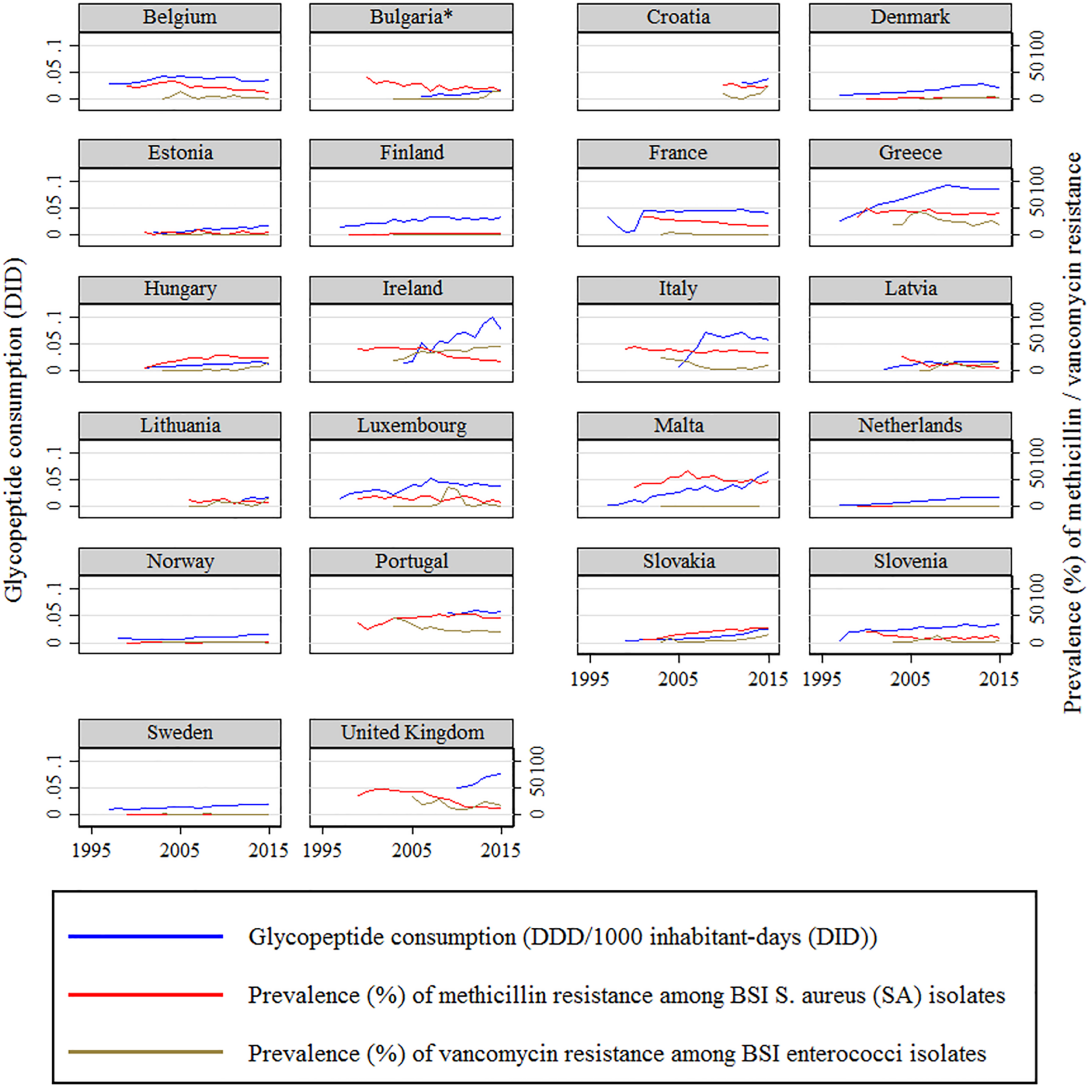
Trends of rates of methicillin resistance among Staphylococcus aureus isolates and rates of vancomycin resistance among enterococci isolates from bloodstream infection (BSI) and glycopeptides use expressed as Defined Daily Dose per 1000 inhabitant–days (DID) for European countries during 19-year period (1997 to 2015).

**Fig 2:**
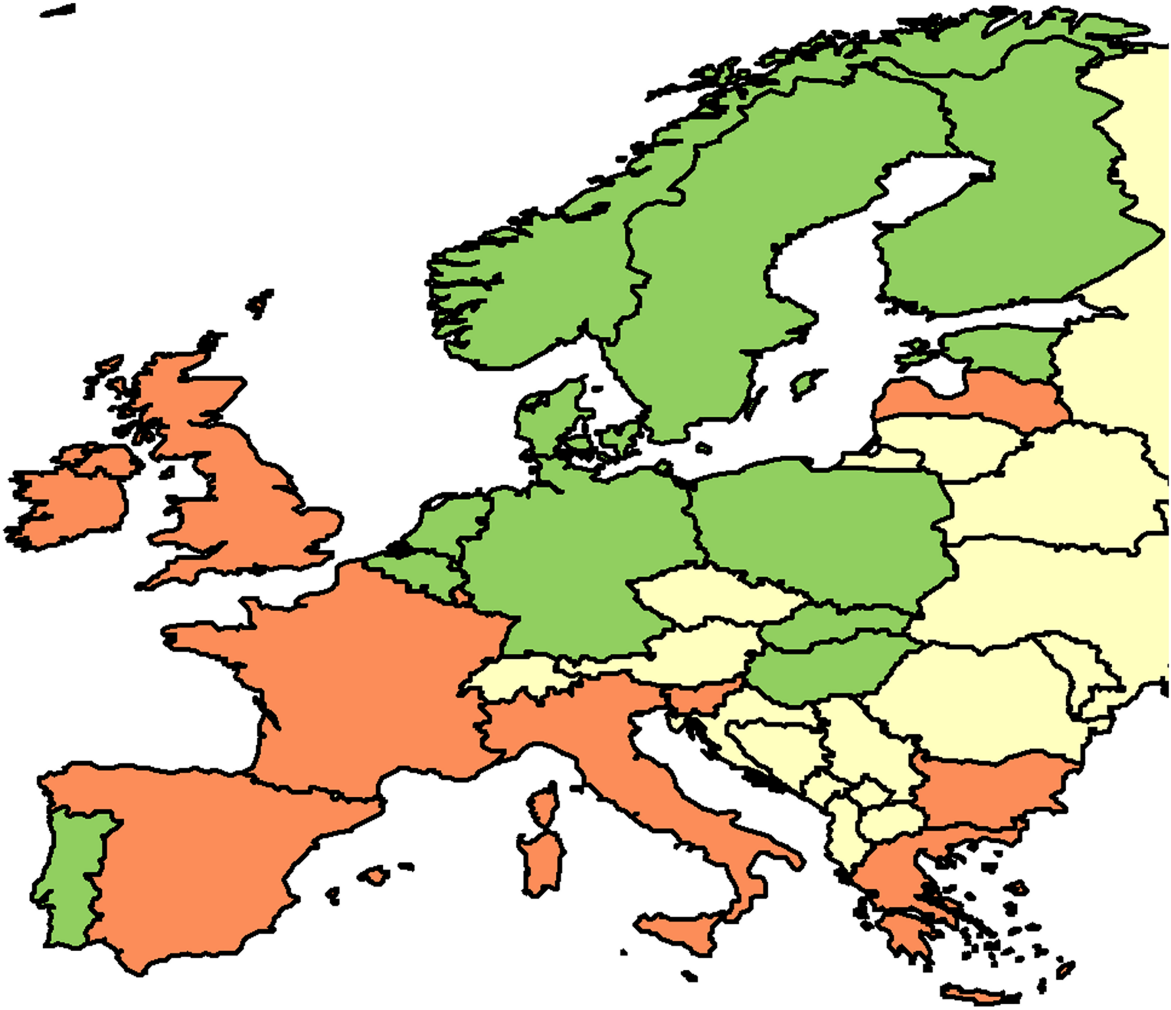
Concordant (green countries) and discordant (red countries) trends in MRSA BSI rates and glycopeptides use adjusted for trend in CoNS and VRE BSI rates in Europe in the study period. The colours represent the European countries with **Red:** discordant trend between glycopeptide consumption and prevalence rate of BSI due to MRSA, VSE and CONS / a concordant trend with a difference of more than 100% between the relative increase in glycopeptide consumption and relative increase in prevalence rates; Discordant trend defined as opposing trends between between glycopeptide consumption and prevalence rate of BSI due to MRSA, VSE and CONS with a difference of more than 50% in relative increase / relative decrease; **Green:** concordant trend between the glycopeptide consumption and prevalence rates of BSI; **Yellow:** No information available.

Since MRSA rates and glycopeptides data were available throughout the study period for the majority of the European countries (21/31), these data were used for the modelling. Fig 1 shows the MRSA, VRE and glycopeptides’ DID trends. To account for the gap in the publication and dissemination of the yearly resistance data to the medical community, a 2-year lag in the resistance rates was applied. Mixed-effect modelling did not show a relationship between glycopeptides use and MRSA rates (RC -0·005, p = 0·136). Similarly, modelling of glycopeptides use and VRE rates did not show any association (RC 0·003, p = 0·613). To account for the variation in resistance rates, European countries were stratified according to the entity of the reduction / increase in the MRSA rates in the study period. In the 8 countries with more than 5% decrease of MRSA BSI, no positive correlation could be observed, rather a negative correlation, with glycopeptides’ DID (RC -0·009, p = 0·059) reflecting the contrasting trend between MRSA and glycopeptides. Fig 2 shows the concordance and discordance of MRSA and glycopeptides’ DID trends after adjusting for VRE and MRCoNS rates data. Strikingly contrasting trends of MRSA rates and glycopeptides DID were observed in 8 European countries.

## Discussion

Our study revealed that between 2005 and 2015, yearly glycopeptides use in European countries significantly increased irrespective of the decreased trend in rates of BSIs due to MRSA and VSE and stable trend of those due to MRCoNS (most important targets for these antibiotics) in the same countries.

The dramatic increase in antimicrobial resistance in gram negative bacteria recently urged the medical community to promote the implementation of antimicrobial stewardship programmes in hospitalised and community patients and to ask for new antibacterial drugs, to require enhanced infection control measures in high risk hospitalised patients[21]. Of great concern is the increased mortality rate reported in patients with severe infections and inappropriate antibiotic therapy [22–24] that ranged from 11% to 72% of treated patients. A gold standard to increase rationale antibiotic usage and implement antimicrobial stewardship programmes however does not exist. Different approaches have been suggested with heterogeneous results including dedicated hospital teams, continuous education, antibiotic formulary, rapid diagnostic tests, decision support systems, and periodic audits[21]. Interestingly, in all these approaches, availability of updated surveillance data are the *“sine-qua-non”* condition to improve the coverage of the most likely infecting microorganism. For this reason in the last decades major stakeholders at national and international levels made an enormous effort to increase national coverage and representatives of surveillance systems of healthcare associated and antimicrobial resistant infections.

The recent NICE[25] and IDSA[8] guidelines for antibiotic stewardship programmes recommend that knowledge of the local AMR rates be taken into consideration for the empiric therapy. However, although the AMR rates in European countries are updated yearly and disseminated widely by the ECDC and by national surveillance systems, our findings suggest an incorrect perception of the rates by the physicians leading to overprescription. A number of potential explanations exist. First, fear of the clinical consequences of antibiotic-resistant infections and a lack of effective point-of-care diagnostics for BSIs may lead to an increase in prescription of wide-spectrum antibiotics in empiric therapy. Lack of de-escalation protocols incorporating microbiological results as soon as they are available may also add to the burden of inappropriate therapy. It is also worthy to note that a high percentage of antibiotic use is in patients with no final microbiological diagnosis, therefore increasing the risk of inappropriate therapy.

The 2005 EARS-Net surveillance report[14] showed a decrease in MRSA BSIs in hospitalized patients for the first time. This change has been attributed partly to the success of infection control programs, although, because of the high heterogeneity in infection control procedures in European countries, major drivers of reduction were difficult to define.[16] The increase in vancomycin use had already been observed in 1984–1996 data from US and European markets.[26] Increase in vancomycin use in countries with decreasing MRSA rate had been observed in Japan[27], France[28], and Hong Kong.[29] Recently, Grau et al[20] analyzed the association between the consumption of anti-MRSA antibiotics and incidence of MRSA in 54 acute care hospitals in Catalonia from 2007 to 2012 and reported a steady increase in the vancomycin use despite a stable MRSA burden. Data from German ICUs over a period of nine years (2001 to 2009)[30] showed an overall increase in the use of antibiotics effective against MRSA although the incidence rate and the proportion of MRSA did not change significantly over time. Conversely, a US study[31] of treatment of MRSA wound infections showed that the proportion of the variation of antimicrobial (linezolid, clindamycin, trimethoprim-sulfamethoxazole, and cephalexin) use was explained by the national and local MRSA burden. Major limitations of the studies rely on the lack of accounting for confounders (e.g., VSE incidence and regional or country data). To reduce these limitations and improve evidence on surveillance impact on prescribing, we designed a study that analyses 31 European countries over a 16-year period and include multiple microrogansims’ resistance data to cover all antibiotic (in our case glycopeptides) therapeutic targets.

The study has limitations. First, the ecological design and the heterogeneity of sources increase the risk of information biases, internal validity, and confounding (i.e. case mix of patients or usage of glycopeptides for the treatment of severe infections due to other drug-resistant Gram-positives not included in our epidemiological analysis). Regarding the information bias, ESAC network uses sources based on national sales and reimbursement data, including information from national drug registries. Although 70% of the hospital data sources in 2012 used standardised operative procedures to homogenize and validate case ascertainment and definitions, sampling and laboratory procedures, an over- or under-estimation of resistance rates is still possible. ECDC reports a high coverage of the total hospitalised population based on their data sources.[32] Note of worthy, although the study was limited to BSI, there is no data showing an increase of MRSA pneumonia or skin infections in Europe in the last years. Second, there are no surveillance data on sepsis syndromes with no identified micro-organism or for cases where no blood cultures were taken before starting empiric therapy with glycopeptides. Third, antimicrobial consumption data were not adjusted for age and weight. The national/regional data were extracted from retrospective and cohort studies and therefore were potentially subject to inaccuracy and confounding. Fourth, the use of DDD could be associated to intrinsic bias [33], although in this study this measure was used only for trend comparison and not as an outcome measure. It is also important to note that we were limited by the unavailability of MRSA incidence. However, recent data clearly showed a significant downward trend from 2005 onwards [16].

Future research should investigate the behavioural determinants connected with the incomplete usage of the surveillance data when prescribing empiric therapy in hospitalised patients and explore cost-effectiveness of new surveillance tools including automated and semi-automated surveillance, inclusion of patient-level data, creation of network of networks (to assure tracing of resistance spreading), and creation of sentinel-laboratories that in real time could provide alert for new resistance pattern.

## Conclusions

With ongoing intensive efforts to reduce and control the use of antimicrobials in Europe to reduce AMR rates, our finding of increasing trends in glycopeptides use despite decreases in e rates of BSIs due to MRSA, MRCoNS, and VSE are worrisome and seems to suggest a partial consideration of the epidemiological data on resistance when prescribing glycopeptides in hospitalised patients. The results of our study could play a significant role in the discussion of components of antimicrobial stewardship programmes. The increasing local vancomycin use should be therefore carefully evaluated in planning and implementing antimicrobial stewardship programmes. Prospective study addressing confounding factors and clinical patients data need to be planned in order to understand. Reasons for these trends need to be explored and corrective actions necessary for change to be taken. In our opinion a global effort, including major international and national stakeholders, is required to improve understanding of and access to surveillance data by doctors in clinical daily life.

## Supporting information

S1 File Data on bloodstream infections (BSIs) due to methicillin-resistant Staphylococcus aureus (MRSA), methicillin-resistant coagulase negative staphylococci (MRCoNS), vancomycin-resistant enterococci (VRE) and glycopeptides use in Europe. (XLSX)

S1 Table National surveillance systems and European research projects reporting surveillance data on bloodstream infections (BSIs) due to methicillin-resistant Staphylococcus aureus (MRSA), methicillin-resistant coagulase negative staphylococci (MRCoNS), and vancomycin-resistant enterococci (VRE) in Europe. (DOCX)
