# Supplementary material for: Antimicrobial resistance rates in gram-positive bacteria do not drive glycopeptides use
Source: PLoS One. 2017 Jul 20;12(7):e0181358. doi: 10.1371/journal.pone.0181358 (PMC5519079; doi:10.1371/journal.pone.0181358)
Supplement: S1 Table — (DOCX) [file pone.0181358.s002.docx]

**S1 Table. National surveillance systems and European research projects reporting surveillance data on bloodstream infections (BSIs) due to methicillin-resistant Staphylococcus aureus (MRSA), methicillin-resistant coagulase negative staphylococci (MRCoNS), and vancomycin-resistant enterococci (VRE) in Europe**

| Surveillance system | Acronym | Country | Coverage |
| --- | --- | --- | --- |
| European Antimicrobial Resistance Surveillance Network[1] | EARS-Net | Europe | 30 European countries |
| European Surveillance of Antimicrobial Consumption[2] | ESAC | Europe | 30 European countries |
| Hospital acquired infections surveillance system (Krankenhaus-Infektions-Surveillance-System)[3] | KISS | Germany | 200 hospitals |
| Austrian Report on Antimicrobial Resistance[4] | AURES | Austria | 40 laboratories |
| English surveillance programme for antimicrobial utilisation and resistance[5] | ESPAUR | England | 160 national acute-care hospitals |
| Observatoire National de l’Épidémiologie de la Résistance Bactérienne aux Antibiotiques[6] | ONERBA | France | 15 microbiological networks |
| Antimicrobial surveillance program[7] | SENTRY | Worldwide | Medical centers and outpatient facilities in more than 30 countries |
| Spanish national health care-associated infection surveillance network[8] | INCLIMECC | Spain | 61 hospitals |
| MRSA regional surveillance network, Twente/Münsterland[9] | EUREGIO MRSA-net | Germany and Netherlands | 40 hospitals |
| Study of Prevalence of Nosocomial Infections[10] | EPINE | Spain | 250 hospitals |
| Surveillance of Health Care Associated Infections in Catalonia[11] | VINCat | Catalona (Spain) | 54 hospitals |
| Spanish national nosocomial infection surveillance network[12] | VICONOS | Spain | 43 public hospitals |
| Surveillance Project for Antibiotic Use in German Acute Care Hospitals (Bunderverhand Deutscher Krankenhaus Apotheker e.V) [13]^37^ | RKI-if-ADKA^37^ | Germany | 109 acute-care hospital centres |

References:

1. European Antimicrobial Resistance Surveillance Network (EARS-Net) [Mar 15, 2017]. Available from: <http://ecdc.europa.eu/en/activities/surveillance/EARS-Net/Pages/index.aspx>.

2. Antimicrobial consumption interactive database (ESAC-Net) [Mar 15, 2017]. Available from: <http://ecdc.europa.eu/en/healthtopics/antimicrobial_resistance/esac-net-database/Pages/database.aspx#sthash.YyVICnnv.dpuf>.

3. Nationales Referenzzentrum für Surveillance von nosokomialen Infektionen (ITS-KISS) [updated Dec 21, 2015Oct 01, 2016]. Available from: <http://www.nrz-hygiene.de/surveillance/kiss/its-kiss>

4. European Centre for Disease Prevention Control. AURES – the first Austrian report on antimicrobial resistance – perspective of the human sector 2007 [ Oct 01, 2016]. Available from: <http://www.eurosurveillance.org/ViewArticle.aspx?ArticleId=3329>.

5. English surveillance programme for antimicrobial utilisation and resistance (ESPAUR). Report 2014 Public Health England; 2014 [updated Nov 18, 2015 Oct 01, 2016]. Available from: <https://www.gov.uk/government/publications/english-surveillance-programme-antimicrobial-utilisation-and-resistance-espaur-report>.

6. Observatoire National de l’Epidémiologie de la Résistance Bactérienne aux Antibiotiques (ONERBA) : rapport d’activité 2011-12 / Annual Report 2011-12. [ Oct 01, 2016]. Available from: [www.onerba.org](http://www.onerba.org).

7. Bell J, Turnidge J. SENTRY Antimicrobial Surveillance Program Asia-Pacific region and South Africa. Communicable Diseases Intelligence Quarterly Report. 2003;27 Suppl:S61-6.

8. Perez CD, Rodela AR, Monge Jodra V, Quality Control Indicator Working G. The Spanish national health care-associated infection surveillance network (INCLIMECC): data summary January 1997 through December 2006 adapted to the new National Healthcare Safety Network Procedure-associated module codes. Am J Infect Control. 2009;37(10):806-12. doi: 10.1016/j.ajic.2009.03.005. PubMed PMID: 19560231.

9. Friedrich AW, Daniels-Haardt I, Köck R, Verhoeven F, Mellmann A, Harmsen D, et al. EUREGIO MRSA-net Twente/Münsterland--a Dutch-German cross-border network for the prevention and control of infections caused by methicillin-resistant Staphylococcus aureus. Euro Surveill. 2008;13(35).

10. Garcia-Cenoz M, Chamorro J, Vidan J, Lanzeta I, Lameiro F, Urtasun JM, et al. [Prevalence of nosocomial infection in Navarre. Aggregated data of the EPINE study for 2005]. An Sist Sanit Navar. 2007;30(1):89-99. PubMed PMID: 17491611.

11. Trilla A. The VINCat Program: quality and safety improvements in Catalonia. Editorial. Enferm Infecc Microbiol Clin. 2012;30 Suppl 3:1-2. doi: 10.1016/S0213-005X(12)70088-5. PubMed PMID: 22776146.

12. Jodra VM, Diaz-Agero Perez C, Sainz de Los Terreros Soler L, Saa Requejo CM, Dacosta Ballesteros D, Quality Control Indicator Working G. Results of the Spanish national nosocomial infection surveillance network (VICONOS) for surgery patients from January 1997 through December 2003. Am J Infect Control. 2006;34(3):134-41. doi: 10.1016/j.ajic.2005.10.004. PubMed PMID: 16630977.

13. Kern WV, Fellhauer M, Hug M, Hoppe-Tichy T, Först G, Steib-Bauert M, et al. Antibiotika-Anwendung 2012/13 in 109 deutschen Akutkrankenhäusern. DMW-Deutsche Medizinische Wochenschrift. 2015;140(23):e237–e46.
